# T and B Cells in Periodontal Disease: New Functions in A Complex Scenario

**DOI:** 10.3390/ijms20163949

**Published:** 2019-08-14

**Authors:** C.M. Figueredo, R. Lira-Junior, R.M. Love

**Affiliations:** 1 School of Dentistry and Oral Health, Griffith University, Queensland 4222, Australia; 2 Menzies Health Institute Queensland, Griffith University, Gold Coast, QLD 4222, Australia; 3 Division of Oral Diseases, Department of Dental Medicine, Karolinska Institutet, 141 04 Stockholm, Sweden

**Keywords:** lymphocyte, T cells, B cells, cytokine, periodontal disease

## Abstract

Periodontal disease is characterised by a dense inflammatory infiltrate in the connective tissue. When the resolution is not achieved, the activation of T and B cells is crucial in controlling chronic inflammation through constitutive cytokine secretion and modulation of osteoclastogenesis. The present narrative review aims to overview the recent findings of the importance of T and B cell subsets, as well as their cytokine expression, in the pathogenesis of the periodontal disease. T regulatory (Treg), CD8^+^ T, and tissue-resident γδ T cells are important to the maintenance of gingival homeostasis. In inflamed gingiva, however, the secretion of IL-17 and secreted osteoclastogenic factor of activated T cells (SOFAT) by activated T cells is crucial to induce osteoclastogenesis via RANKL activation. Moreover, the capacity of mucosal-associated invariant T cells (MAIT cells) to produce cytokines, such as IFN-γ, TNF-α, and IL-17, might indicate a critical role of such cells in the disease pathogenesis. Regarding B cells, low levels of memory B cells in clinically healthy periodontium seem to be important to avoid bone loss due to the subclinical inflammation that occurs. On the other hand, they can exacerbate alveolar bone loss in a receptor activator of nuclear factor kappa-B ligand (RANKL)-dependent manner and affect the severity of periodontitis. In conclusion, several new functions have been discovered and added to the complex knowledge about T and B cells, such as possible new functions for Tregs, the role of SOFAT, and MAIT cells, as well as B cells activating RANKL. The activation of distinct T and B cell subtypes is decisive in defining whether the inflammatory lesion will stabilise as chronic gingivitis or will progress to a tissue destructive periodontitis.

## 1. Introduction

Periodontal disease is an inflammatory response to bacterial biofilm accumulation around teeth. This host inflammatory response is mediated mainly by neutrophils, monocytes/macrophages, and T and B lymphocytes (T and B cells). The development of the disease is characterised by a dense inflammatory infiltrate in the connective tissue, in which polymorphonuclear leukocytes and macrophages are abundant immune cells that firstly respond to the bacterial insult. When inflammation is not resolved, antigen-presenting cells (APCs) are activated by bacterial products and interact with naive T helper cells (Th0), driving their differentiation into several subsets, such as Th1, Th2, Th9, Th17, T-follicular helper (Tfh), and regulatory T cells (Treg) [1]).

Overexpression of the Th17/Treg axis is seen in disease initiation, followed by persistence of the Th17 response in periodontitis progression in a non-human primate model of periodontitis [2]. Tregs are required to keep periodontal health homeostasis, where their presence is essential to ensure a controlled response that minimises collateral tissue damage [3]. However, recent evidence suggested Th17 might not be the main source of interleukin (IL)-17A in periodontal tissues, suggesting Tregs may have a more prominent role in the pathogenesis of the periodontal disease [4]. These findings might change our understanding of the current literature. More recently, a new cell type named mucosal-associated invariant T (MAIT) cells has been associated with autoimmune and other inflammatory diseases in humans [5] and its role in the periodontal disease pathogenesis should be taken into consideration.

B cells, on the other hand, are part of the adaptive humoral immunity specialised in secreting antibodies and cytokines, as well as presenting antigens, and have been strongly associated with periodontal homeostasis and disease [5]. A minimal presence of B cells in healthy gingiva has been reported [6,7], which might be important to avoid bone loss due to the subclinical inflammation that occurs in the clinically healthy periodontium. B cells in periodontitis patients may contribute to chronic systemic inflammation through cytokine secretion [8]. Memory B cells can induce bone loss in rheumatoid arthritis [9], which led to the hypothesis that they express RANKL and regulate alveolar bone homeostasis during periodontitis [10]. The present narrative review aims to overview the recent findings of the importance of T and B cell subsets, as well as their cytokine expression, in the pathogenesis of the periodontal disease.

## 2. T and B Lymphocytes

### 2.1. T Lymphocytes

T lymphocytes (T cells) are immune cells involved in host defence and control of immune-mediated inflammatory disease development. They can be distinguished from other lymphocytes by the presence of a T-cell receptor (TCR) on the cell surface. Most T cells are composed of two glycoprotein chains named α (alpha) and β (beta) TCR chains. However, a smaller group of T cells, named γδ T cells, presents a gamma/delta composition. They are an important subset of T cells as they can recognise a broad range of antigens without the presence of major histocompatibility complex (MHC) molecules [11]. T cells are subdivided into Th, Treg, T cytotoxic (CD8^+^), natural killer, and memory cells. Also, T cell anergy has been defined as a mechanism of peripheral tolerance that determines the functional inactivation of T cells following antigen recognition under non-optimal conditions [12].

Naive CD4^+^ T cells are activated after interaction with antigen-MHC complex and differentiate into specific subtypes depending on the cytokine microenvironment [13]. After such interactions, they become activated and differentiated into effector T cells, which are responsible for the production of effector molecules, such as pro-/anti-inflammatory cytokines and cytotoxic molecules. Most of the effector T cells undergo programmed cell death after the antigen clearance. The ones that survive differentiate into memory T cells. Th cells are classified as Th1, Th2, Th17, and Treg subpopulations based on their unique cytokine properties [14].

Besides the classical Th cell subpopulations, Tfh, Th9, and Th22 cells have recently been defined as new subpopulations that produce IL-21, IL-9, and IL-22, respectively [14,15]. Tfh is a specialised CD4^+^ T-cell subset that provides survival, proliferation, and selection signals by engaging in cognate interactions with B cells [16]. Th9 cells have been shown to present both beneficial and detrimental functions. Among the beneficial functions is their ability to initiate antitumor immunity and immune response to helminth parasites [17]. Detrimental functions of Th9 cells include the promotion of allergic inflammation and the mediation of some types of autoimmunity [17]. Duhen et al. [18] showed that memory T cells with skin-homing properties from healthy donors are characterised by the production of IL-22 in the absence of IL-17 and IFN-γ. The authors found that several T cell clones isolated from psoriatic lesions were Th22. Their function is the specific production of IL-22, which has been linked to skin homeostasis and inflammation [14].

Cytotoxic T lymphocytes (CD8^+^ T cells) are generated in the thymus and express a dimeric co-receptor, usually composed of one CD8α and one CD8β chain, and they recognise peptides presented by MHC class I molecules. CD8^+^ T cells present three major mechanisms to kill infected or malignant cells: (a) secretion of cytokines, primarily TNF-α and IFN-γ, which have anti-tumor, anti-viral, and anti-microbial effects, (b) production and release of cytotoxic granules (also found in NK cells), which contain two families of proteins, perforin and granzymes, and (c) destruction of infected cells via Fas/FasL interactions which can result in apoptosis of the target cell. Besides cytotoxic activities, CD8^+^ T cells also have regulatory/suppressor functions (CD8^+^ Treg), since they can control other leukocytes to avoid excessive immune activation and its pathological consequences [19].

Besides the T cells subgroups mentioned above, mucosal-associated invariant T (MAIT) cells represent a unique subset of innate-like T cells described in the late 1990s [20]. MAIT cells represent the most abundant innate-like T-cell population within human beings, comprising up to ~5% of the total T-cell population [21]. Characterisation of the MAIT cells in the buccal mucosa showed that the major subset displayed a tissue-resident and activated profile with high expression of CD69, CD103, HLA-DR, and PD-1. These tissue-resident MAIT cells produced higher IL-17 levels than tissue non-resident and circulating populations [22]. New research projects should be stimulated to better understanding the role of tissue-resident MAIT cells in the osteoclastogenesis activation in periodontal diseases.

### 2.2. B Lymphocytes

B lymphocytes (B cells) are part of the humoral component of the adaptive immune system and specialised in secreting antibodies. B cells can also present antigens and secrete cytokines. In mammals, B cells mature in the bone marrow, and B cell receptors (BCRs) mature on their cell membranes, which allow B cells to bind specific antigens initiating an antibody response. Such antigen recognition can happen through either low- or high-affinity binding modes [23]. After maturation, multiple subsets of B-cells co-expressing IgM and IgD emerge from the bone marrow and colonise compartments of secondary lymphoid organs [24].

B cell subpopulations can be distinguished in peripheral blood based on surface-marker expression, which mainly represents different developmental stages of the cell. Alterations in some of these populations have been associated with clinical phenotypes in immunodeficiency and autoimmune diseases. The second phase of B-cell development occurs after the antigen-dependent phase. Depending on various contacts and cytokine stimuli received by the activated cell, it will become either a memory cell to be activated once again in the future or it will become a plasma cell producing large amounts of antibodies [25].

Didactically, B cells can be subdivided as follows: plasmablasts (the immature precursor of plasmacytes), plasma cells (antibody-secreting cells arising from B cell differentiation), memory B cells (B2 cells and synonymous with classical B cells), marginal zone (MZ) B cells (specialized population of B cells that are located in the marginal zone of the spleen), B1 cells (subtype of B cells that are distinct from classical B cells with respect to their phenotype, distribution in the body, and function). Unlike classical B cells, B-1 cells are considered functionally to be part of the innate immune response [26]. Regulatory B cells (Breg) negatively regulate the immune response by producing regulatory cytokines and directly interacting with pathogenic T cells via cell-to-cell contact [27].

Abnormal B-cell recognition of self-antigens may lead to autoimmunity, which results in autoantibody production. Autoantibodies produced by B-cell-derived plasma cells provide diagnostic markers for autoimmunity, and also contribute significantly to disease pathogenesis [28]. Autoimmune diseases where B-cell functions are closely correlated with disease activity include systemic lupus erythematosus, rheumatoid arthritis, scleroderma, type 1 diabetes, and multiple sclerosis [28].

### 2.3. T and B Lymphocytes in Periodontal Homeostasis

#### 2.3.1. T Lymphocytes

The characterisation of T lymphocytes in healthy gingiva has shown a dominance of CD4^+^ helper T cells [6]. These cells play fundamental roles in the adaptive immune responses, and their cytokine production in response to specific immunological challenges led to the classical framework of distinct Th cell subsets [29]. CD8^+^ T cells were the second most abundant T lymphocyte in healthy gingiva, followed by a small percentage of γδT cells. Gingival CD8^+^ T cells seem to have regulatory/suppressor properties important to the maintenance of gingival tissue integrity by downregulating inflammation under homeostatic conditions. These cells can produce IL-10 and TGF-β, which then suppress osteoclastogenesis [30]. Tissue-resident epithelial γδ T cells have been reported to be the major T cell population in the epithelial tissues and are important in carrying out barrier surveillance and helping to keep tissue homeostasis, and to some extent, epithelial repair [31]. Gingival γδ T cells accumulate after birth in response to barrier damage and are crucial for immune homeostasis. These cells produce amphiregulin, a wound healing-associated cytokine, which limits the development of periodontitis [32]. γδ T cells are also the major source of IL-17 in homeostasis. Interestingly, ablation of γδT cells resulted in increased gingival inflammation and alterations in the microbial diversity [33]. Within the CD4 compartment, about 15% are presumed to be Treg cells, which are crucial for periodontal homeostasis. Increased numbers of Tregs are associated with bone homeostasis, even in the presence of local inflammation [34] and may be related to the non-progression of gingivitis lesions in some patients, even after a long period of oral biofilm exposure. The new roles of γδT cells and Tregs in periodontal tissue homeostasis are crucial to the understanding of periodontal disease initiation and progression.

Dutzan et al. [6] characterised memory and naive T-cell subsets in the gingiva, showing that approximately 80% of CD4^+^ and 50% of CD8^+^ T cells had a CD45RO^+^ (activated T cell) memory phenotype. The CD4^+^ cell compartment in gingiva had a minimal CD45RA^+^ (naive T cell) population, but the CD8^+^ T cell compartment had a substantial population of CD45RA^+^/CCR7^−^ cells (terminally differentiated effector T cells, T_EMRA_) alongside a smaller population of naive CD45RA^+^CCR7^+^ cells [6,35]. CCR7 is a chemokine receptor that divides human memory T cells into two functionally distinct subsets. CCR7^−^ memory cells express receptors for migration to inflamed tissues and display immediate effector function; CCR7^+^ memory cells lack immediate effector function, but efficiently stimulate dendritic cells and differentiate into CCR7^−^ effector cells upon secondary stimulation [36].

The combination of CD45RO and CCR7 (memory subset markers) with CD69 (a stimulatory receptor expressed at sites of chronic inflammation) was performed by Dutzan et al. [6] to analyse circulating and tissue-resident memory CD4^+^ and CD8^+^ T cell subsets. Their results showed that the majority of CD4 memory T cells in gingiva were CCR7^−^CD69^+^ (resident effector memory, rT_EM_ cells), followed almost equally by resident memory, effector memory (T_EM_), and central memory (rT_CM_; CCR7^+^CD69^+^) cells. Regarding CD8, memory CD45RO^+^ cells were also rT_EM_ in their majority, followed by a large population of T_EM_ and a small population of central memory cells (T_CM_). Increased proportions of resident memory T cells are common at barrier sites, where they have been reported to support early/immediate defence mechanisms, providing site-specific protection from pathogen challenges [37]. Resident memory T cells would then have special importance to protect the connective tissue form the bacterial products released in the periodontal sulcus.

Besides the T-cell characterisation, it is important to understand how these cells behave under physiological conditions. Having clinically healthy gingiva does not ensure that T cells are not being activated. Dutzan et al. [38], demonstrated in mouse gingiva that gingiva-resident Th17 cells developed via a commensal colonisation-independent mechanism. Th17 cells might accumulate at the gingiva in response to the physiological barrier damage that occurs, for instance, during mastication. The authors showed that physiological mechanical damage could induce the expression of IL-6 from epithelial cells, promoting an increase in gingival Th17 cell numbers.

#### 2.3.2. B Lymphocytes

The characterisation of B cell subsets in gingival tissues was recently described by Mahanonda et al. [39]. The authors reported very few naïve B cells (<8%) in all stages of healthy and diseased tissues. Additionally, memory B cells (CD19^+^CD27^+^CD38^−^) represented the majority of the B cell population in the clinically healthy gingiva and were detected in the connective tissue subjacent to the apical region of the junctional epithelium, which could be due to the local low-grade inflammatory response to a constant challenge of the biofilm. The authors highlighted the importance of detecting memory B cells in clinically healthy human gingiva since very little is known about memory B cells residing in human nonlymphoid tissues. The minimal presence of B cells in healthy gingiva was also reported by others [6,7,35,40]. Such low levels might be important to avoid bone loss around teeth due to the subclinical inflammation that occurs in the clinically healthy periodontium.

Another aspect of the B cell biology that is relevant for gingival homeostasis is the production of antibodies against periodontal pathogens, which can contribute to host protection [41,42]. Page et al. [43] demonstrated that immunisation using *P. gingivalis* as antigen could reduce the onset and progression of alveolar bone loss in non-human primates. Also, Shelburne et al. [41] suggested that anti-*P*. *gingivalis* HtpG antibodies predict health in patients susceptible to periodontal disease. The potential role of B cell humoral immunity in maintaining homeostasis needs further investigations. A description of the main functions of T and B cells’ subsets in the periodontal tissues is presented in the Table 1.

### 2.4. Changes with Age

Furthermore, age is also a variable that needs to be considered when evaluating the lymphocyte function in healthy periodontium. The effects of aging on periodontal tissues are thought to intensify alveolar bone resorption in elderly individuals [44]. Witkowski et al. [45] reviewed the proteodynamics in aging human T cells and reported that the proteolytic elimination of altered proteins, as well as modulation of the activity of those remaining, leads to the dynamic change of proteome composition and function in aging lymphocytes. Ebersole et al. [44] reported that several B cell and plasmacyte genes are altered in aging healthy gingival tissues, which are mainly associated with antigen-dependent activation and B cell differentiation/maturation processes. Aging T and B cell dynamics requires further comprehensive analysis and may influence the pathophysiology of periodontal disease.

### 2.5. T and B Lymphocytes in Periodontal Inflammation

In 1983, Okada et al. [46] published a very elegant paper characterising the immunocompetent cells on histological sections from diseased human gingiva. According to the authors, human periodontitis contains numerous sets of infiltrating cells which are organized unusually, with a region rich in T lymphocytes and monocytes/macrophages just subjacent to the pocket or sulcular epithelium; and a region in the central lamina propria, located farther away from the microbial agent, which is rich in B cells and plasma cells and poor in T lymphocytes. Furthermore, the same group characterised the T lymphocyte subsets (T4+ and T8+) in the inflamed gingiva from human periodontitis and showed the ratio T4+/T8+ was lower in gingival tissue than in peripheral blood [47]. Dutzan et al. [6] evaluated the major cell subsets and revealed that the lymphocytic compartment, CD3+T cells remained the dominant population in both health and disease, yet in disease the total number of T cells is much greater, reflecting a 10 fold increase in total inflammatory cells [6].

The role of T cells in the immune dysregulation of periodontitis has been consistently revised by Campbell et al. [1]. Activated Th1, Th2, and Th17 cells can produce a variety of pro-inflammatory cytokines, such as IL-1β, IL-17E (IL-25) and IL-17, that activate other immune cells such as dendritic cells, neutrophils, and B cells. Activation of both T cells and subsequently, B cells can cause the production of the receptor activator of nuclear factor κ B -Ligand (RANKL), which leads to alveolar bone resorption by osteoclasts, resulting in tooth loss. Moreover, the activation of B cells by Tfh in either peripheral lymph nodes or tertiary lymph organs can result in clonal activation of B cells, which produce antibodies to recognise bacterial components; however, production of autoantibodies to collagen, fibronectin and laminin can contribute to local destruction of the gingival tissue. Finally, a lack of Treg cells or an inability of those present to reduce local inflammatory responses by other immune cells may play a role in the chronic inflammation associated with periodontitis [1].

T cells have a crucial role in the tissue secretion of IL-17, a cytokine strongly associated with bone loss around teeth. Chen et al. [48] reported that IL-17 and IFN-γ levels in biopsy specimens of gingival lesions from chronic periodontitis patients were higher than those in the healthy controls. Moreover, relative IFN-γ, IL-17A, and T-bet mRNA levels were also significantly higher in patients with chronic periodontitis compared to controls, suggesting that Th17 and Th1 cells might be involved in the pathogenesis of chronic periodontitis. Dutzan et al. [6,35] characterised IL-17-secreting cells within the hematopoietic compartment in healthy and periodontitis gingival samples and found a significant increase in IL-17^+^ cells in diseased sites. The major source of IL-17 was CD4^+^ T cells, with minimal contribution from CD8, γδT, and non-T-cell sources.

Moreover, the percentage of CD4^+^ T cells producing IL-17 significantly increased in periodontitis. CD4^+^ T cells preferentially upregulated IL-17 and not IFN-γ in gingival tissue from periodontitis patients. The same group also reported that Th17 cells in periodontitis are dependent on the local dysbiotic microbiota, and both IL-6 and IL-23 are required for their accumulation. Also, pharmacologically targeting RORγt, a transcription factor relevant for Th17 differentiation, reduces alveolar bone loss in a murine model of periodontitis [49]. On the other hand, Parachuru et al. [4] presented an interesting paper that compared healthy/gingivitis tissues with chronic periodontitis tissues. Among other goals, they aimed to determine the identity of FoxP3 and IL-17A positive cells in periodontal tissues. The authors reported that Th17 cells either do not exist in periodontal disease or are present in small numbers and that, as with other chronic inflammatory lesions, the source of the relatively small amounts of IL-17 may be mast cells. Moreover, they also suggested that Tregs may have an important role in the pathogenesis of the chronic inflammatory periodontal disease. Such a statement needs to be better investigated since it might affect our present understanding of the Th17/Treg imbalance that leads to periodontal disease progression.

Besides the importance of IL-17 secreted by CD4^+^ T cell or mast cells, a novel T cell-secreted cytokine, called secreted osteoclastogenic factor of activated T cells (SOFAT), that can induce osteoclastogenesis in a RANKL-independent manner, has been described in periodontal tissues [50]. The authors showed that the mRNA and protein levels of SOFAT were significantly higher in the gingival tissue of periodontitis patients compared to controls. More recently, Jarry et al. [51] demonstrated that B-lineage cells, including plasma cells, also exhibited strong staining for SOFAT in diseased periodontal tissue. Therefore, SOFAT might have an important role in periodontal disease by activating RANKL related osteoclastogenesis.

The characterisation and identification of interstitial T cells are relevant to understanding the immunopathogenesis of periodontitis. Bittner-Eddy et al. [52] have made an important contribution to this subject by describing a flow cytometry assay that distinguishes interstitial leukocytes in the oral mucosa of mice from those circulating within the vasculature or in post-dissection contaminating blood. They reported that, unlike circulating CD4 T cells, interstitial CD4 T cells were almost exclusively antigen-experienced cells (CD44^hi^). The authors reported the presence of antigen-experienced *P. gingivalis*-specific CD4 T cells in nasal-associated lymphoid tissues following oral feeding of mice with *P. gingivalis.* Such differentiation might be critical for future understanding of the players driving alveolar bone destruction.

B cells infiltrate and dominate sites showing progressive chronic inflammatory periodontal disease in humans [53]. It has been shown that periodontitis lesions contain significant numbers of immunoglobulin-bearing lymphocytes and plasma cells, suggesting that the clinical progression of the periodontal lesion is followed by a shift in cellular infiltrates from predominantly immunoglobulin-negative lymphocytes to IgG and IgM-bearing lymphocytes and plasma cells [54]. Oliver-Bell et al. [55] demonstrated that B cells make a substantial contribution to alveolar bone loss in murine periodontitis, probably due to B-cell activation and expression of RANKL in the gingiva. Abe et al. [56] reported that ligature-induced periodontitis resulted in significantly less bone loss in B cell-deficient mice compared with wild-type controls, supporting the importance of B cells in periodontal bone loss. The authors also suggested that two cytokines of the TNF ligand superfamily, a proliferation-inducing ligand (APRIL) and B-lymphocyte stimulator (BLyS), might be potential therapeutic targets in periodontitis [56].

Mahanonda et al. [39] have characterised B cell subsets in gingivitis and periodontitis. The density of memory B cells in periodontitis lesions was significantly lower than in healthy and gingivitis tissues. On the other hand, Ab-secreting cells were the major cell type in the CD19^+^ B cell population, with the mean percentage of Ab-secreting cells being significantly higher than that of memory B cells. Moreover, an abundance of CD138^+^ plasma cells was observed in periodontitis tissues. The authors reported that plasma cells were arranged in clusters detected at the base of the periodontal pocket area and scattered throughout the gingival connective tissue, especially apically toward the advancing front of the lesion [39].

B cells in patients with periodontal disease may contribute to chronic systemic inflammation through constitutive secretion of IL-8 and IL-1β [8], but the in situ impact of such cytokine production should be elucidated. Kawai et al. [57] have demonstrated that B cells can be the cellular source of RANKL for bone resorption in homogenised gingival tissue from sites showing periodontal disease. Moreover, Malcolm et al. [58] have shown that the percentage of B cells expressing RANKL was elevated following *P. gingivalis* infection in gingival tissues. Oliver-Bell et al. [55] have also investigated the impact of *P. gingivalis* infection in the RANKL expression of B cells, showing that mice infected with *P. gingivalis* presented a significant increase in B-cell RANKL expression in the gingiva. Moreover, B-cell-deficient mice did not show *P*. *gingivalis*-induced alveolar bone loss. Recently, Kanzaki et al. [59] demonstrated that sRANKL and TNF-α cleaved from activated tumour necrosis factor-α-converting enzyme-bearing B cells might be important as an osteoclastogenic factor in periodontitis lesions.

Han et al. [10] suggested that B cells affect alveolar bone homeostasis in a murine model of periodontitis through antibody-independent and RANKL-dependent mechanisms. They reported that gingival memory B cells promote osteoclastogenesis and that this potential was increased by the development of periodontitis. Demoersman et al. [60] reported that a significantly higher percentage of CD27^+^ memory B cells was observed in patients with severe periodontitis. At the same time, human B1 cells, which were previously associated with a regulatory function, decreased in such patients. The authors also reported that the RANKL expression increased in every B cell subset from severe periodontitis patients and was significantly greater in activated B cells than in the subjects without periodontitis. Moreover, an interesting literature review published by Zouali [54] supports that B cells are key participants in RANKL-mediated bone resorption. Activated RANKL-positive B cells can exacerbate alveolar bone loss in a RANKL-dependent manner in animal models. On the other hand, blocking RANKL, B-cell-activating factor (BAFF), and a proliferation-inducing ligand (APRIL) reduces alveolar bone loss in experimental models of periodontitis. Coat et al. [61] reported that periodontal parameters could be significantly improved after treatment with rituximab, concluding that anti-B lymphocyte therapy could be beneficial in improving de clinical conditions of patients with periodontitis.

**Figure 1:**
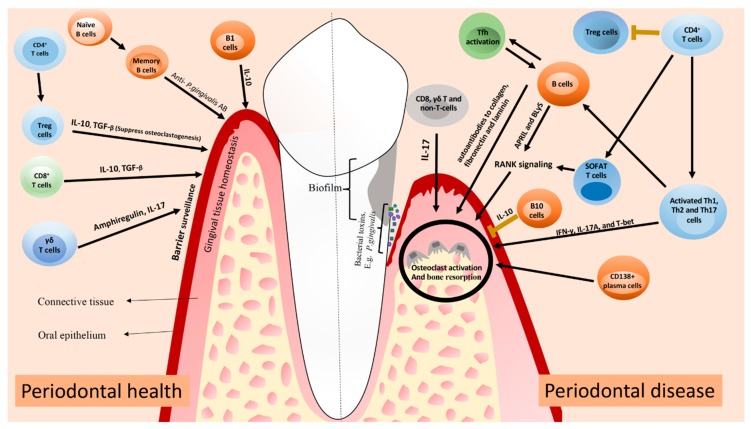
A summary of how mentioned T and B cells can contribute to periodontal health and disease. In periodontal health, Treg and CD8+ T cells contribute to periodontal homeostasis through the production of IL-10 and TGF-β. γδ T cells produce amphiregulin and IL-17 to promote periodontal homeostasis. B cells produce antibodies against periodontal pathogens, limiting the development of periodontal inflammation. In periodontal disease, activated Th1, Th2, and Th17 cells produce pro-inflammatory cytokines that contribute to tissue damage. Both T and B cells produce RANKL, which leads to osteoclast activation and alveolar bone resorption. Clonal activation of B cells by Tfh cells can lead to the production of autoantibodies to collagen, fibronectin and laminin, contributing to local tissue destruction. Lack of Treg cells or an impaired function probably impact on the development of periodontitis. IL-17 produced by other cells can also contribute to tissue damage via osteoclast activation. The figure was adapted from Lira-Junior & Figueredo [67].

Besides the fact that B cells positively activate immune responses, functioning as APCs and producing antibodies, regulatory B cells (Bregs) have been shown to exert a suppressive role in immune response [62]. B10 cells are a Breg cell subset that produces IL-10 and therefore, negatively regulates the inflammatory responses [63]. B10 cells are present in gingival tissues of patients with and without periodontal disease, but in significantly higher levels in periodontal disease lesions [5.89 ± 2.02) when compared to healthy tissues (0.1 ± 0.3, *p* < 0.01) [64]. Yu et al. [65] demonstrated that the local induction of IL-10 competency of B10 cells was associated with the inhibition of both inflammation and bone loss in ligature-induced experimental periodontitis. Wang et al. [66] also reported that the adoptive transfer of B10 cells significantly inhibited inflammation and bone loss in a mouse model of experimental periodontitis, suggesting a potential novel principle of treatment for periodontal diseases. It has been suggested that the in vitro treatment of B10 cells with a combination of IL-21, anti-Tim1, and CD40L might inhibit periodontal bone loss in ligature-induced experimental periodontitis [65]. A schematic of the lymphocyte subsets and their possible contribution to periodontal homeostasis and inflammation is presented in the Figure 1.

The increased knowledge in T and B cells’ biology, as well as their functions during gingival tissue homeostasis and inflammation might help in designing novel therapeutic strategies for bone disorders where these cells are a crucial part of the local tissue inflammation, such as periodontitis.

## 3. Conclusions

Several new roles have been discovered and added to the complex knowledge about T and B cells. These include different functions of Tregs, the role of γδ T cells in gingival homeostasis, the role of SOFAT in osteoclastogenesis, and the possible pathogenic role of MAIT cells. Also, the importance of B cells activating RANKL-mediated bone resorption adds to this complex scenario. During periodontal inflammation, the activation of distinct T and B-cell subtypes, as well as their cytokine production, are crucial in defining whether the inflammatory lesion will stabilise as chronic gingivitis or progress to tissue-destructive periodontitis.

## Figures and Tables

**Table 1:** A summary of the main functions of mentioned T and B cells in periodontal health and disease.

Cell	Subtype	Function in the Periodontal Tissues
**T cells**	Treg	Periodontal homeostasis by producing IL-10 and TGF-β.
	MAIT	Largely unknown.
	Th	Specific immunological challenges lead to distinct cells’ subsets; Th1, Th2, and Th17 cells can produce a variety of pro-inflammatory cytokines that activate other immune cells such as dendritic cells, neutrophils, and B cells. Th17 can also be produced in response to biological barrier damage in healthy tissue.
	Tissue-resident epithelial γδ	Barrier surveillance, tissue homeostasis, and epithelial repair; Major source of IL-17 in homeostasis.
	CD8^+^	Downregulate inflammation and suppress osteoclastogenesis. IL-10 and TGF-β production.
	Tfh	Activation of B cells; IL-21 production.
	SOFAT	Induce osteoclastogenesis in a RANKL-independent manner.
**B cells**	Activated	Activation and expression of RANKL in the gingiva; promote osteoclastogenesis.
	Memory	To prevent bone loss due to subclinical inflammation in clinically healthy periodontium. Production of antibodies against periodontal pathogens.
	Immunoglobulin-bearing lymphocytes; plasma cells	Clinical progression of the periodontal lesion; Stimulate the expression of RANKL in the gingiva.
	B1	Associated with regulatory functions and the numbers might be decreased in periodontitis patients; produces antibodies against antigens and act as antigen-presenting cells.
	Breg/B10	Negatively regulates the inflammatory responses via IL-10.
	CD138^+^ plasma cells	Association with the advancing front of the periodontal lesion.

Treg: regulatory T cells; MAIT cells: mucosal-associated invariant T cells; Th: helper T cells; CD: cluster of differentiation; SOFAT: secreted osteoclastogenic factor of activated T cells; Tfh: T-follicular helper; Breg: regulatory B cel.
